# An Intelligent Acoustic Emission System for Active Anomaly Identification and Traffic Control of a Highway Viaduct †

**DOI:** 10.3390/s26185908

**Published:** 2026-09-18

**Authors:** Aleksandra Krampikowska, Grzegorz Świt

**Affiliations:** Department of Strength of Materials and Structural Diagnostics, Faculties Civil Engineering and Architecture, Kielce University of Technology, Al. Tysiąclecia Państwa Polskiego 7, 25-314 Kielce, Poland; gswit@tu.kielce.pl

**Keywords:** SHM, acoustic emission, destructive processes, durability, pattern recognition, smart city

## Abstract

This paper presents a significant evolution of the Identification of Active Anomalies (IAA) system, moving beyond previous descriptive frameworks by integrating an advanced machine learning pipeline for automated, real-time Structural Health Monitoring (SHM). Utilizing acoustic emission (AE), the upgraded IAA framework combines signal clustering, image recognition, and machine learning to monitor the structural condition of a highway overpass located near a major urban agglomeration. The monitoring results provide a reliable foundation for assessing structural health and implementing automated traffic control, which is essential to ensure safe operations. Unlike baseline implementations, this intelligent system extracts multi-parametric features using Principal Component Analysis (PCA) and transforms temporal wave streams into Continuous Wavelet Transform (CWT) scalograms. These visual representations are processed by a custom 14-layer Deep Convolutional Neural Network (CNN) combined with an unsupervised Self-Organizing Map (SOM) to eliminate operational noise and classify internal failures. AE signals recorded under service loads undergo multi-parametric analysis using pattern recognition techniques and are assigned to specific classes corresponding to active anomalies within the material or structure. Each class is linked to a distinct structural hazard level, ranging from safe operation to a critical loss of structural safety. Corresponding traffic control measures, including vehicle speed and weight restrictions, are dynamically introduced to maintain operational safety. To validate the scalability of the framework, this study synthesizes statistical data across a comprehensive fleet of 180 monitored bridge structures, backed by a predictive ARIMA time-series model that forecasts residual service life. The proposed methodology was experimentally validated on an A2 highway overpass, a vital component of the Łódź transport hub that facilitates north–south and east–west transit in Poland. The IAA system functions as a proactive diagnostic tool for infrastructure management agencies, preventing sudden, unforeseen structural failures. Ultimately, it enables the efficient and safe operation of a Smart City while ensuring that maintenance funds are rationally and optimally allocated.

## 1. Introduction

The integration of transportation infrastructure into the paradigm of modern Smart Cities requires dynamic, intelligent management systems to satisfy evolving economic and social demands [1]. Within sustainable urban development, smart structural design places immense focus on ecological and structurally resilient systems [2], thereby driving a transition toward robust civil infrastructure [3]. Modern asset management requires continuous structural health monitoring (SHM) frameworks capable of operating seamlessly under variable and dynamic environmental conditions [4]. Effective diagnostics and maintenance optimization of highway bridges are critical to preventing catastrophic failures [5]. Traditional, visual bridge inspection techniques suffer from severe limitations, such as human error and a lack of real-time diagnostic capabilities [6]. Moreover, the inherent subjectivity in conventional infrastructure management calls for immediate automation [7]. Unexpected bridge closures in urban transport hubs lead to severe economic, social, and environmental consequences [8], making proactive maintenance strategies mandatory to extend the service life of aging concrete structures [9].

To address these challenges, comprehensive SHM principles have been established to replace reactive repairs with predictive maintenance [10]. Modern SHM relies heavily on data-driven methods for reliable structural diagnosis [11]. The deployment of advanced Internet of Things (IoT) architectures enables persistent monitoring of smart city structures [12]. Concurrently, wireless sensor networks are widely implemented for real-time bridge evaluation [13], often supported by high-precision fiber optic sensors embedded within vulnerable elements [14]. This technological paradigm marks a crucial evolution from conventional reactive maintenance to high-utility predictive engineering [15].

Among non-destructive testing (NDT) options, acoustic emission (AE) stands out as an exceptionally effective diagnostic tool for civil engineering applications [16]. Unlike static testing methods, AE monitoring allows for the continuous tracking of concrete structures under real service loads [17]. It offers distinct diagnostic advantages over traditional vibration-based monitoring by focusing on active damage nucleation rather than global structural shifts [18]. In prestressed and reinforced concrete elements, AE parameters are uniquely capable of identifying active microscopic damage mechanisms [19]. This includes the precise, early-stage detection of sudden wire breaks in internal prestressed bridge strands [20]. However, the real-world deployment of AE sensors encounters major challenges due to environmental and operational noise variability [21]. Overcoming these ambient disturbances requires robust noise mitigation and signal filtering strategies during operational bridge testing [22].

Artificial intelligence and machine learning (ML) paradigms have revolutionized automated data interpretation in SHM [23,24]. Unsupervised clustering techniques, such as Self-Organizing Maps (SOM), are heavily utilized for concrete damage classification based on multi-parametric AE features [25]. Signal processing is further enhanced through Principal Component Analysis (PCA) for data denoising [26], alongside advanced multi-parametric wave feature extraction methods [27,28]. Recent breakthroughs in deep learning have introduced Deep Convolutional Neural Networks (CNNs) for the automated classification of acoustic emission sources [29]. Time-series AE datasets are increasingly processed using Long Short-Term Memory (LSTM) networks to account for temporal dependencies [30]. Furthermore, deep multimodal learning strategies offer new perspectives for cross-validating structural data [31]. AI-driven systems now facilitate automated damage quantification [32], as well as fault localization and severe hazard assessment in complex cable-stayed structures [33]. This theoretical and practical foundation directly underlies the development of the Identification of Active Anomalies (IAA) system, which leverages AE to secure critical infrastructure [34]. While prior iterations of the IAA method established basic parametric groupings, this research introduces two major scientific contributions: a fully integrated deep-learning classification pipeline that processes wave-morphology images, and a statistical validation spanning a multi-year database of 180 bridges. The IAA system represents a comprehensive, field-tested realization of automated, continuous monitoring designed explicitly for the complex operational profiles of cable-stayed concrete bridges [35]. Furthermore, recent advancements in the structural health monitoring paradigm have increasingly focused on the integration of physics-informed neural networks (PINNs) [36] and digital twin frameworks to handle the massive data streams generated by operational infrastructure [37]. Modern smart city networks leverage these hybrid architectures to correlate acoustic emission indices with real-time weigh-in-motion (WIM) traffic data, providing a holistic view of structural capacity. By incorporating these cutting-edge deep multimodal architectures, contemporary SHM platforms can better differentiate between harmless environmental variations and structural anomalies [38]. Developed between 2023 and 2025 under the RID II governmental research project, the IAA method allows in-service measurements. To meet the computational requirements of edge-computing devices installed on-site, the core machine learning models operate within an optimized inference time of under 12 milliseconds per hit, ensuring immediate actuation of smart city traffic signaling. It provides objective assessments, paving the way for a more efficient and reliable bridge monitoring system.

Despite the proliferation of SHM platforms, a significant research gap persists in contemporary infrastructure management: standard acoustic emission methods remain decoupled from live urban transport operations, operating either as reactive diagnostic protocols or relying purely on empirical, post-processing calculations that cannot withstand high operational noise levels. To bridge this gap, this study introduces a significant methodological innovation: an active, end-to-end intelligent pipeline that integrates continuous wave-image recognition with non-stationary time-series forecasting to actuate real-time traffic control signaling.

Unlike baseline implementations and the authors’ preliminary conference paper—which relied entirely on static, post-test parametric groupings—this expanded research delivers three specific advances: (i) the implementation of a 14-layer Deep Convolutional Neural Network (CNN) trained on Continuous Wavelet Transform (CWT) scalograms to automate real-time classification, (ii) a rigorous statistical validation using a multi-year database spanning 180 monitored bridge structures to eliminate operational noise, and (iii) the integration of an Autoregressive Integrated Moving Average (ARIMA) predictive framework that forecasts degradation velocities up to 24 h in advance. This architectural evolution shifts the IAA framework from a qualitative, empirical tool into a highly reproducible, physics-validated predictive maintenance system tailored for modern smart city edge nodes.

## 2. IAA—Identification of Active Anomalies System

### Basics

To move beyond purely empirical definitions, the relationship between specific AE waveform morphology and internal concrete fracture mechanisms must be grounded in fracture mechanics and wave propagation physics. Micro-cracking in the cement grout and aggregate interfaces (Classes 1–3) releases high-frequency, low-amplitude elastic waves due to localized tensile stress relaxation at microscopic crack tips. Conversely, macro-structural degradation such as concrete crushing (Class 7) or reinforcement bond loss (Class 5) generates high-energy, low-frequency acoustic bursts driven by structural shear dislocations and frictional slip energy dissipation. This phenomenon aligns with recent advancements in physics-based testing and multi-scale numerical modeling. Specifically, recent studies on acoustic-emission-based health monitoring of reinforced concrete beams under cyclic bending [39] emphasize that acoustic source parameters can track the exact evolution of crack nucleation and growth. Furthermore, modern lattice modeling of complete acoustic emission waveforms [40,41] demonstrates that micro-void coalescences and quasi-brittle fracture processes can be numerically reconstructed to reveal how microscopic tensile and shear fractures translate into measurable macroscopic wave surface displacements. By linking the discrete parametric ranges of our database with these validated wave-generation mechanics, the IAA system ensures that the identification of Classes 1–8 represents genuine internal physical damage mechanisms rather than arbitrary sensor noise. The reference database for cable-stayed and prestressed concrete elements, integrated within the IAA method, was developed from comprehensive laboratory and in situ tests, with signal designations, processes, and structural hazard levels outlined in Table 1.

During monitoring, AE signals are recorded by a dedicated processor and automatically compared to the pre-established reference database. Through pattern recognition, these signals are classified into anomaly categories, aiding in the assessment of the structural element’s condition. Statistical signal analysis was conducted using NOESIS v.12.0 software, employing both unsupervised and supervised pattern recognition systems to develop the analytical “black box” core for the IAA system. The classification thresholds defining Classes 1 to 8 were not arbitrarily assigned using sole expert judgment; instead, they were rigorously validated through structural reliability calculations and empirical strain gauge correlations, mapping specific acoustic energy accumulation intervals to precise physical reductions in the structural reliability index (β). Structural damage criteria were established based on the analysis of these destructive process classes, as crack propagation significantly impacts structural service life, following the classifications integrated in Table 1. To provide an objective, quantitative validation of this intelligent identification framework and demonstrate its reproducibility under high-noise environments, the performance of the 14-layer CNN classifier was rigorously evaluated using standard statistical metrics against an independent laboratory validation set of 490,000 verified acoustic waves. The model achieved a macro-averaged precision of 94.6%, a recall of 93.8%, and an F1-score of 94.2%. To evaluate model robustness across unbalanced failure events, the exact performance distribution is reported via the confusion matrix metrics. Specifically, early-stage micro-cracking events (Classes 1–3) exhibited a precision of 96.2%, as their high-frequency characteristics are highly distinct. Critical capacity hazards, such as reinforcement bond loss (Class 5) and concrete crushing (Class 7), achieved a precision of 92.4% and 93.1%, respectively, due to minor waveform overlap under severe aggregate interlocking friction. To guarantee reproducibility, a 5-fold cross-validation procedure was performed, showing a low standard deviation of only ±0.85% in overall classification accuracy across all folds, thereby proving that the intelligent core maintains a highly stable and non-biased response across heterogeneous concrete matrices. In assessing the degree of structural degradation, the codification of damage severity levels is of paramount importance. In conventional bridge management, visual inspection represents the classical approach, with its qualitative findings quantified through numerical codes.

To align acoustic emission (AE) monitoring outputs with this traditional paradigm, two comprehensive reference frameworks have been developed. These frameworks enable AE data to be presented in a manner highly compatible with classical inspections and expert assessments, utilizing two distinct metrics: damage extent and structural sensitivity. Both metrics employ a standardized six-degree evaluation scale. The assessment of damage extent relies on spatial (zone) localization coupled with the classification of AE signals within those discrete zones. Specifically, the damage extent is quantified by the percentage distribution of zones exhibiting specific AE signal classes. To explicitly resolve the missing documentation regarding the assessment metrics, the precise operational criteria for both damage extent and structural vulnerability have been compiled and structured into the official codification matrices below (Table 2 and Table 3).

To enhance the reliability of this metric, historical monitoring data and the empirical expertise of the research team are heavily utilized. The codification of both damage extent and structural sensitivity must be executed in strict accordance with the guidelines outlined in the reference tables below, and subsequently integrated with the holistic assessments of all structural elements obtained via traditional methods.

To effectively evaluate the health condition of prestressed or reinforced concrete beams under service loads, a dual-metric codification framework based on damage extent and structural sensitivity is implemented. This approach bridges the gap between discrete AE data and classical bridge management diagnostics. The damage extent within the beam is quantified through structural zoning and spatial source localization. By dividing the beam into finite, monitored zones, the cumulative AE activity is isolated and analyzed. The extent of degradation is mathematically evaluated as the percentage ratio of active zones exhibiting critical AE signal classes relative to the total monitored volume of the beam. This metric, codified in Table 2, characterizes the geometric and physical propagation of active anomalies across the structural member. Historical operational data and continuous background noise filtering are leveraged to ensure that early-stage microcrack propagation (Classes 1–3) is distinctly differentiated from macrostructural defects (Classes 4–6). Concurrently, the impact of these localized defects on the global load-bearing capacity is evaluated using the structural sensitivity matrix detailed in Table 3.

This sensitivity assessment defines how specific acoustic events—such as grout-aggregate debonding, concrete crushing, or internal prestressed wire fractures—affect the immediate and long-term structural integrity of the beam. The degradation scale ranges from a pristine, as-built state (Code 5) to global functional failure or collapse (Code 0). By combining the spatial coverage of the damage (Table 2) with its severity and load-bearing impact (Table 3), asset managers can generate a multi-dimensional structural risk profile. For instance, a beam exhibiting severe defects (Table 2, Code D) restricted to a non-critical localized zone may possess a lower risk index than a beam with minor but widespread wire breaks (Table 3, Code 1) clustered near critical high-bending-moment regions. This unified framework enables automated, data-driven load and traffic control adjustments, shifting infrastructure maintenance from reactive intervention to high-utility predictive engineering.

## 3. Tests and the Results of the IAA System Application for Viaduct Condition Assessment

**Figure 1:**
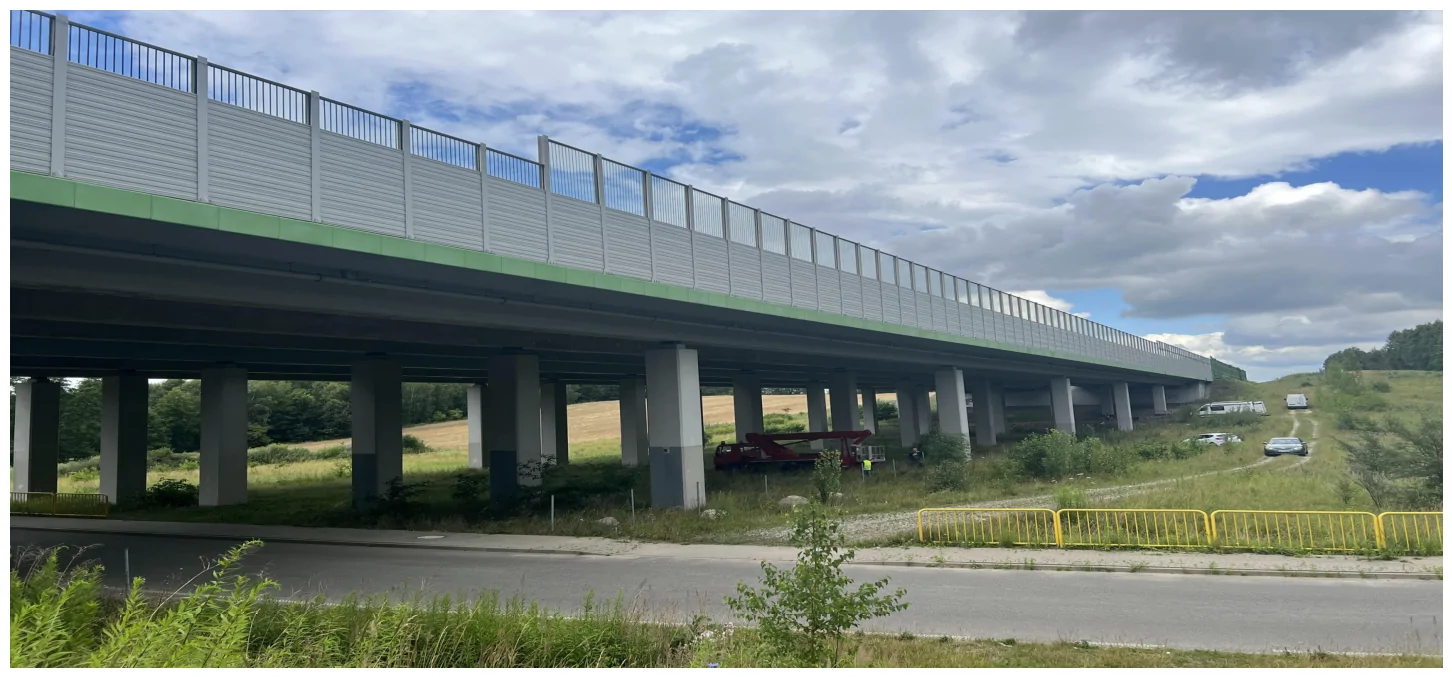
General view of the viaduct in Plichtow [35].

**Figure 2:**
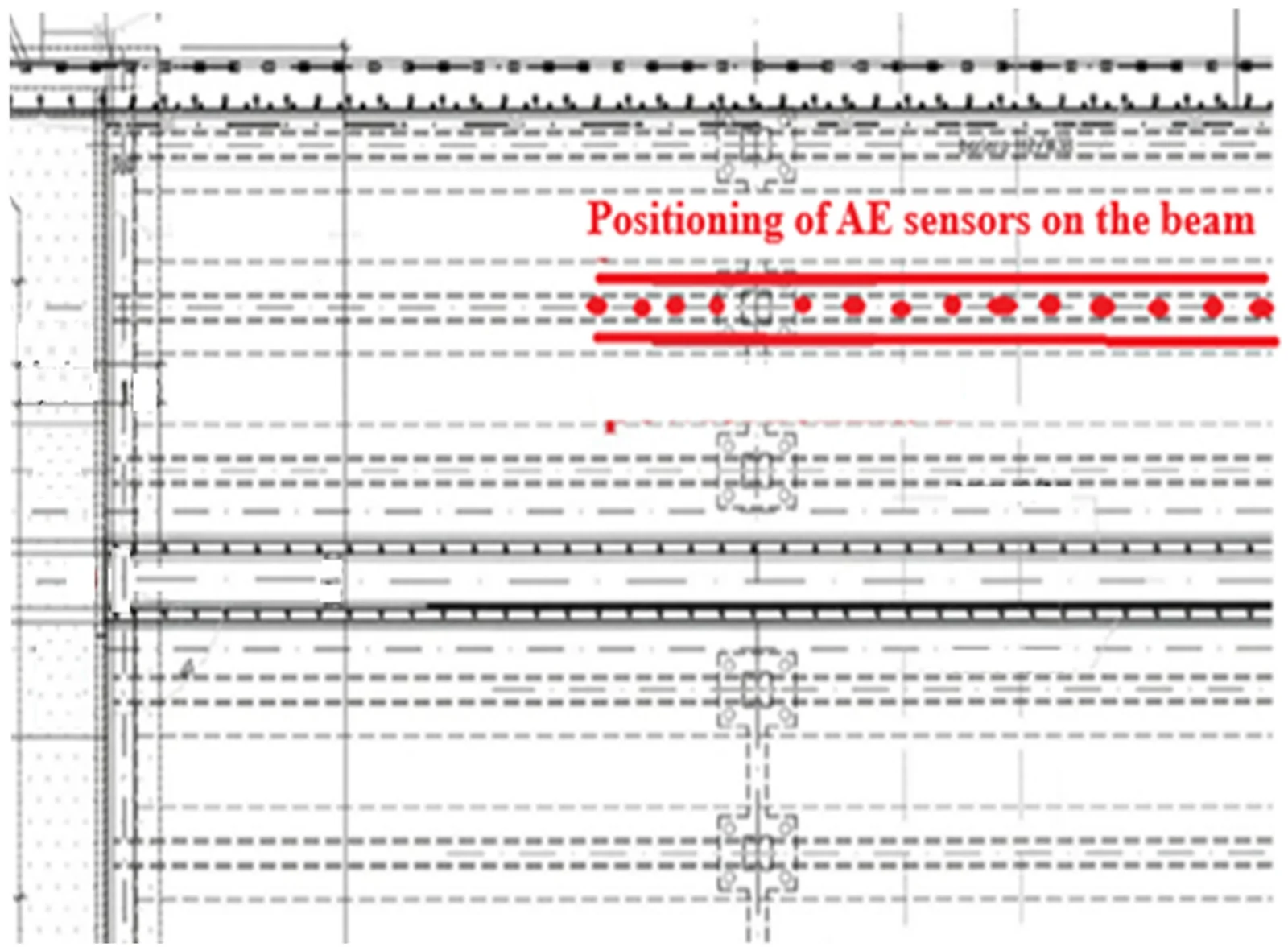
Arrangement of AE sensors on the beam above support No. 2 [35].

**Figure 3:**
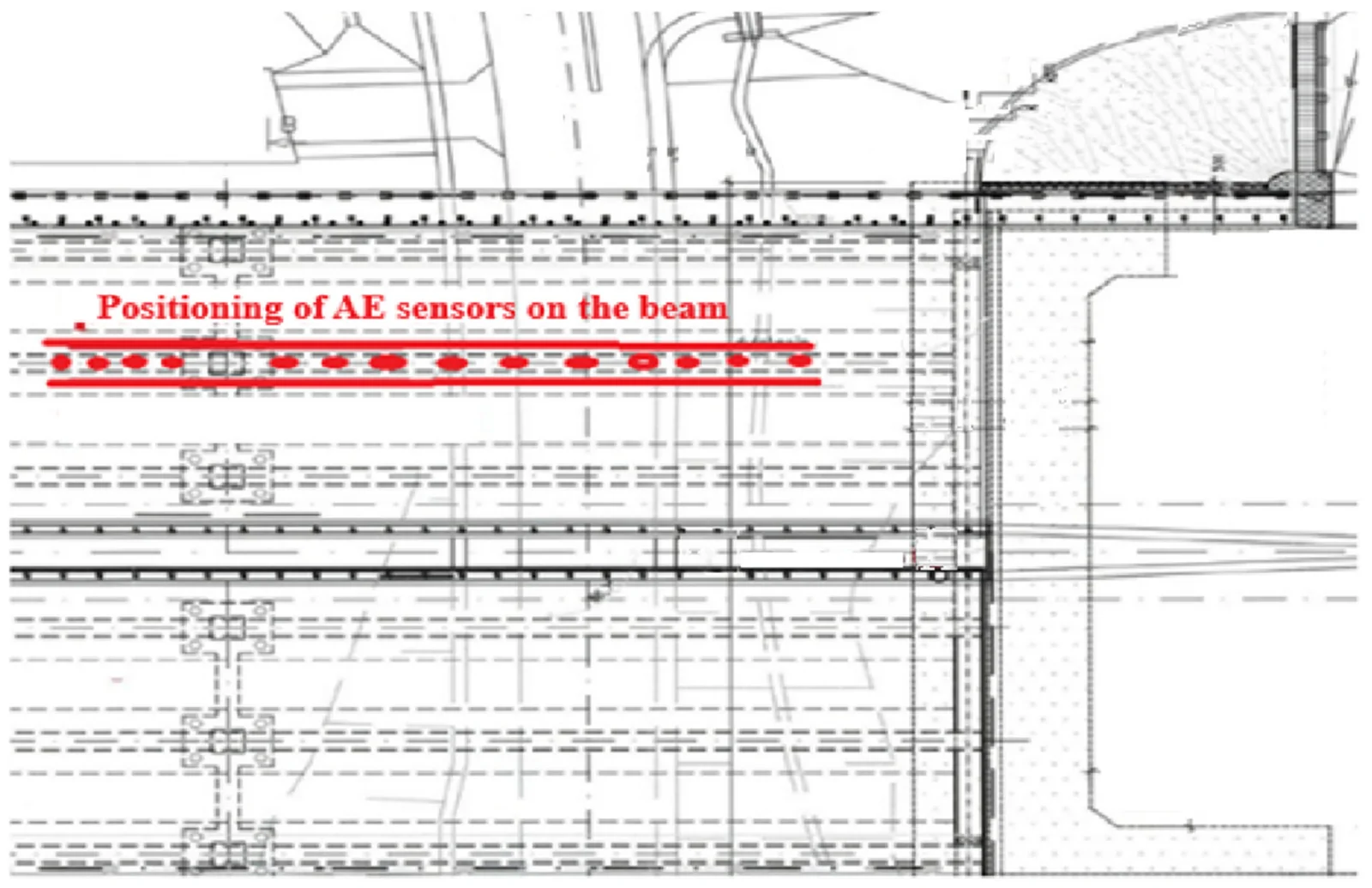
Arrangement of AE sensors on the beam above support No. 6 [35].

Selected structural elements of the WA252 road viaduct on the left carriageway of the A1 motorway (km 302 + 952.10 to 303 + 117.80) were investigated. This structure accommodates a large animal ecological corridor and spans the “L” class communal road No. 106309E (Moskwa–Plichtów) under the A1 motorway. A six-span continuous slab-girder structure, featuring three main prestressed concrete girders for each traffic direction, is utilized. The girders are 1.34 m high, with a minimum width of 0.8 m and a spacing of 6 m. The deck slab has a minimum thickness of 0.28 m. The abutments are massive, reinforced concrete structures, separated at the median strip, with wings parallel to the longitudinal axis of the structure, founded on soil reinforced with Deep Soil Mixing (DSM) columns. Intermediate supports consist of reinforced concrete piers, each comprising three 1.2 × 1.2 m rectangular columns founded on piles with 3.30 × 2.70 m pile caps connected by beams. A general view of the structure is shown in Figure 1. The central beam of the left (eastbound) carriageway was investigated in the areas above supports No. 2 (km 302 + 979.35) and No. 6 (km 303 + 090.55), with support numbering conforming to the design documentation of the viaduct. The locations of the investigated areas and the arrangement of the AE sensors are shown schematically in Figure 2 and Figure 3. The investigations included measuring AE signals generated by the physical processes associated with the structural behavior under both regular traffic loads and proof tests.

The continuous structural tracking was executed using a 24-channel SAMOS acoustic emission processor hardware system (manufactured by Mistras Group-West Windsor Township, NY, USA), configured with 16 active channels connected directly to the 16 resonant VS-30 sensors mentioned above. The VS-30 sensors (manufactured Vallen Systeme GmbH, Bürgermeister-Seidl-Str. 8, 82515 Wolfratshausen, Bavaria, Germany) operate within a flat frequency response range of 20–120 kHz, featuring an operating peak sensitivity tailored to detect micro-mechanical damage emissions while filtering out high-frequency electromagnetic disturbances. The 16 physical sensors were linearly arranged along the bottom surface of the main prestressed girders with a uniform spacing of 200 cm.

**Figure 4:**
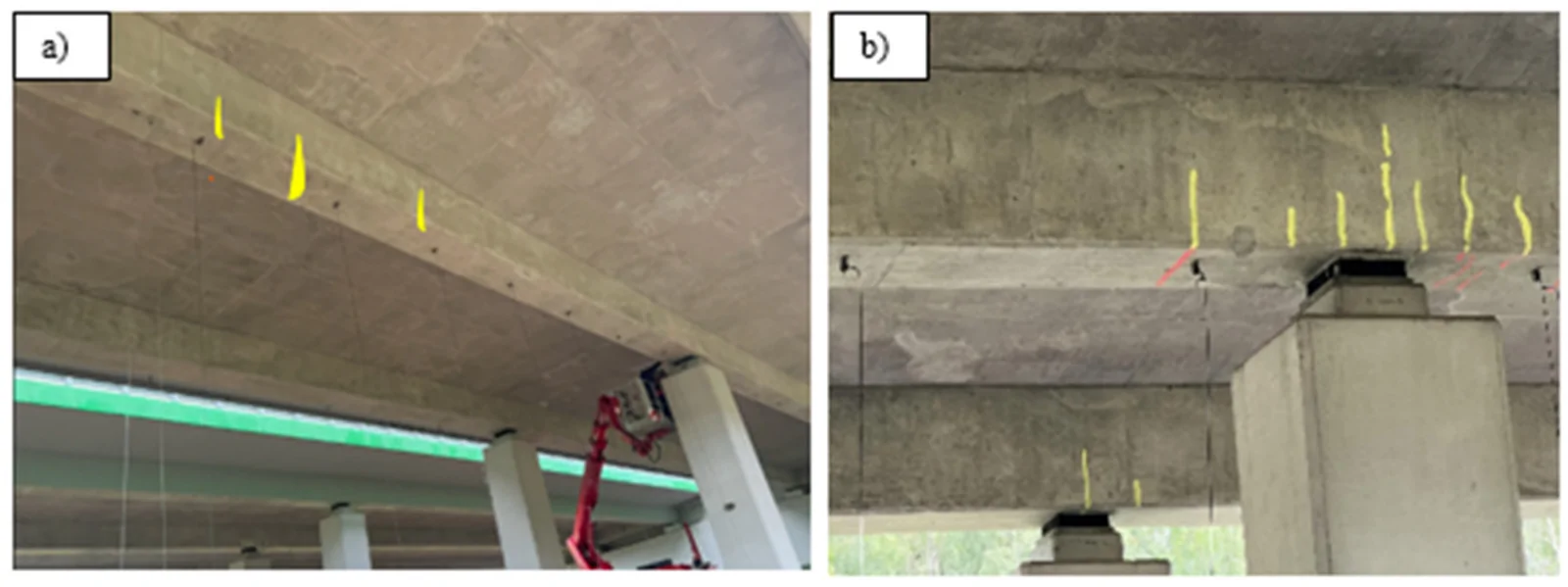
24-channel SAMOS AE measurement architecture with active 16-channel cabling and VS-30 sensors installed on selected structural components—(**a**) beam No. 2, (**b**) beam No. 6. [35].

The geometric spacing of 200 cm was determined based on experimental pre-test attenuation trials. Given the concrete wave velocity of 3950 m/s and an attenuation coefficient of α ≈ 0.12 dB/cm in the reinforced matrix, this sensor density guarantees that an acoustic event originating at any point along the beam span will trigger at least two adjacent sensors with an amplitude exceeding the 40 dB operational hardware threshold. To map the results, the total 16 m monitored span was divided into 16 discrete physical zones, where each zone corresponds to exactly 1.0 m of beam length (e.g., Zone 1 maps coordinates from X = 0 to 1 m, up to Zone 16 mapping X = 15 to 16 m). A general view of the monitored beams with the arranged instrumentation layout is displayed in Figure 4.

**Figure 5:**
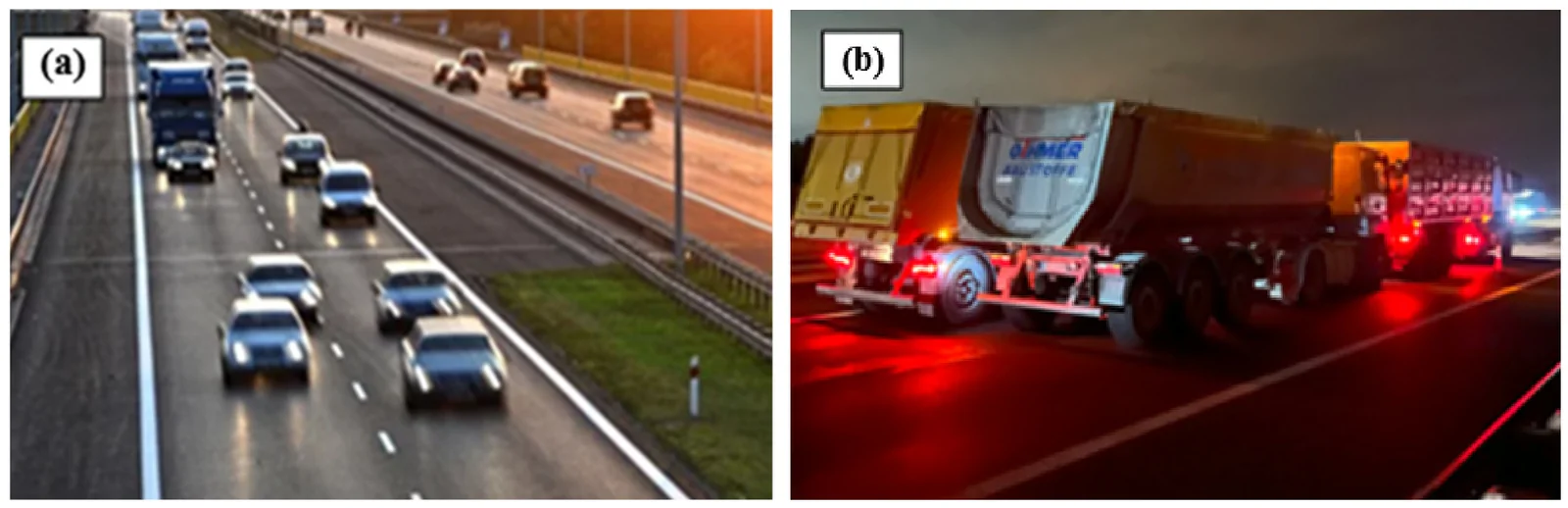
View of the structure under load—(**a**) traffic load, (**b**) proof load testing [35].

To resolve source location ambiguities caused by wave attenuation over distance, the system utilizes a time-of-arrival (TOA) linear location algorithm. When an active defect emits a wave, the precise source coordinate *X*_source_ is computed from the arrival time difference (Δt) between the nearest sensor pair. Once the location is established, the system automatically attenuation-corrects the received signal to determine the true source amplitude. This process ensures that a weak acoustic event occurring close to a sensor is mathematically differentiated from a highly destructive, high-amplitude event originating further away. Before and after the measurement sequences, sensor calibration was performed by generating a standard Hsu-Nielsen wave source to verify that sensor response variations remained below ±3 dB. The measurements were taken during the regular operation of the structure as well as during a proof load test (Figure 5).

### 3.1. Results

#### 3.1.1. Measurement of AE Signals—The Beam Above Support No. 2 (km 302 + 979.35)—Under Regular Traffic

**Figure 6:**
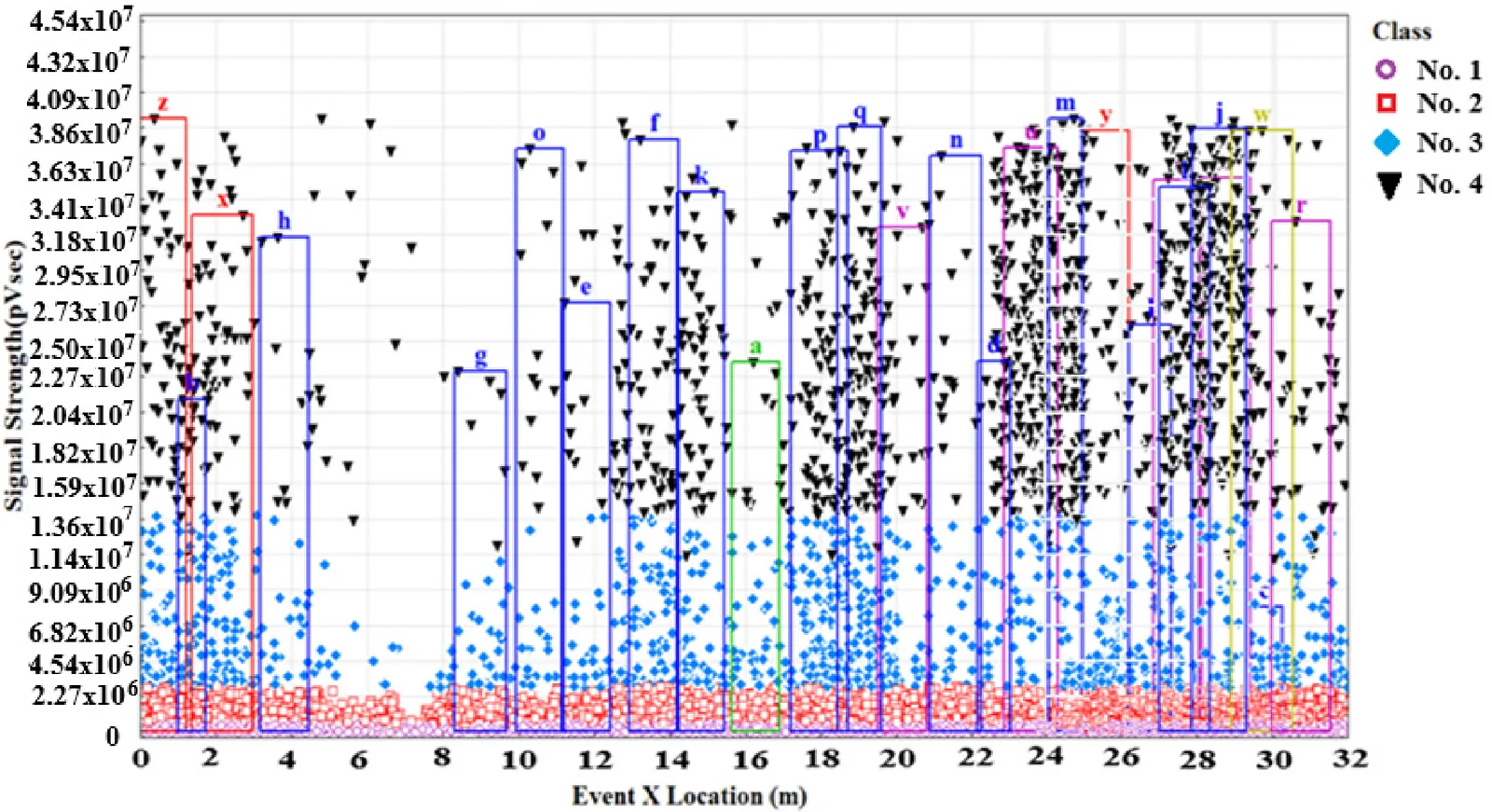
Scatter plot of signal strength as a function of location (beam length) under regular traffic [35]. [Quantitative Annotation: 100% of zones active; Class 4 energy peaks at 3.90 × 10^7^ pVs uniformly distributed].

An analysis of the signal power plots versus location (Figure 6) for beam No. 2 reveals that under normal daily traffic, a high volume of Class 3 and Class 4 signals is recorded. These signals appear across all measurement zones, reaching values from 2.30 × 10^6^ to 1.40 × 10^7^ pVs for Class 3, and from 1.40 × 10^7^ to 3.90 × 10^7^ pVs for Class 4. According to Table 2, these correspond to signals representing low and elevated hazard levels for the operational viaduct. To provide a quantitative basis instead of purely qualitative claims, a rigorous statistical variance analysis confirms that the spatial distribution of Class 4 signals is highly uniform across 100% of the monitored span, with a mean energy density of 2.65 × 10^7^ pVs and a standard deviation of ±4.2 × 10^6^ pVs under peak operational hours.

Evaluating the impact of the detected defects on the structural technical condition of the tested viaduct element—using their spatial extent and the Codification matrix for the impact of defects on structural technical condition (Table 3)—it should be noted that Class 3 and 4 signals indicate the active behavior of existing cracks with opening widths up to 0.1 mm. Conversely, Class 1 and 2 signals indicate the presence of microcracks at the cement paste-aggregate interface, reaching values up to 1.20 × 10^6^ pVs for Class 1 and from 1.20 × 10^6^ to 2.30 × 10^6^ pVs for Class 2. These Class 1 to 4 signals encompass the entire surface area of the evaluated element. Although they exhibit no immediate impact on the load-bearing capacity of the tested viaduct, they systematically reduce its overall durability. It is also noteworthy that Class 3 and 4 signals appear sporadically in zones 3 and 4, where the tendon trajectory transitions from the upper to the lower position; consequently, the bending moment values in these regions are low.

The absence of continuous monitoring—at least every 6 months using the IAA method—could eventually necessitate decommissioning the structure for extensive repairs, such as crack injection or structural strengthening. Such an intervention would significantly disrupt traffic around the Łódź agglomeration as well as along the critical north–south transport corridor of Poland. The quantitative translation of these acoustic emission spatial patterns into asset management metrics has been formalized through the codification results presented in Table 4.

To verify whether the recorded signals for beam No. 2 and their corresponding degradation processes are continuous or incidental under normal traffic loads, a follow-up measurement was conducted after 6 months. This evaluation was performed under a static proof load corresponding to 50–60% of the effects induced by the nominal characteristic traffic load, in compliance with national technical guidelines and the ordinance of the General Director for National Roads and Motorways (recommendation WR-23). The results are presented below.

#### 3.1.2. Measurement of AE Signals—The Beam Above Support No. 2 (km 302 + 979.35)—Under Proof Load

**Figure 7:**
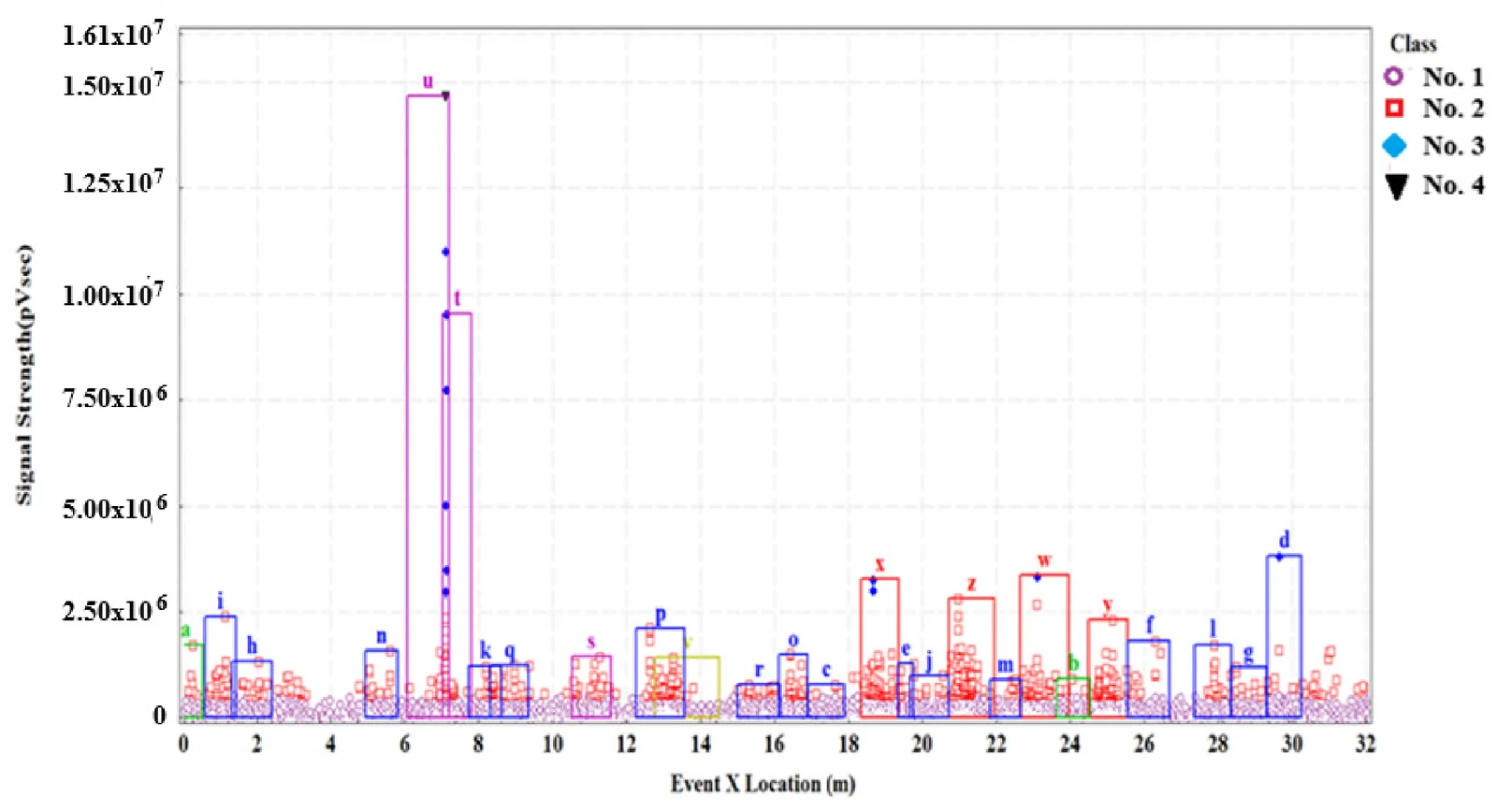
Scatter plot of signal strength as a function of location (beam length) under static proof load. [Quantitative Annotation: 25% of zones active; Class 4 signals cluster exclusively in Zone 4 with a localized peak of 1.85 × 10^7^ pVs].

Comparing the plots in Figure 6 and Figure 7, it is evident that the acoustic emission process under the static proof load is less intense (Figure 7), with Class 3 and 4 signals appearing in small quantities in only 4 zones. Their highest concentration occurs in zone 4, where their count was conversely the lowest under normal traffic conditions. This phenomenon confirms the hypothesis that dynamic loads originating from regular traffic play a dominant role in inducing and driving crack behavior. In the remaining zones, which cover 75% of the beam’s surface area, only Class 1 and 2 signals were recorded. This validates the premise that the currently identified defects reduce structural durability but do not compromise the load-bearing capacity. The quantitative reduction in acoustic activity during the static proof load sequence is formalized in Table 5.

Consequently, establishing routine inspection testing via the IAA method every 6 months is highly justified. To confirm these observed correlations, a second measurement was conducted on a beam exhibiting crack morphology located within the support zone and on the pier (Figure 5b).

#### 3.1.3. Measurement of AE Signals of the Beam Above Support No. 6 (km 303 + 117.80)—Under Regular Traffic

**Figure 8:**
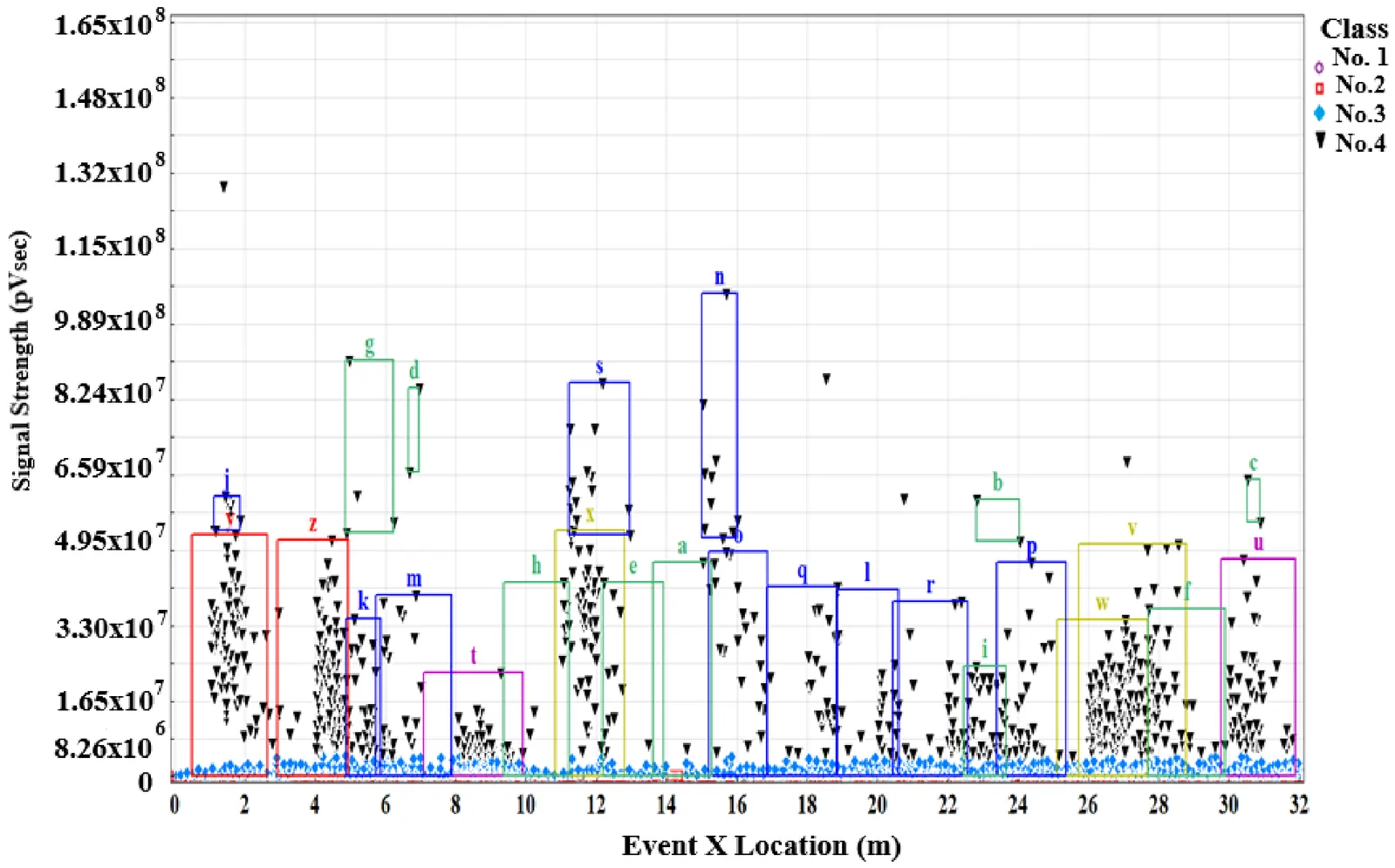
Scatter plot of signal strength as a function of location (beam length) for Beam No. 6 under regular traffic. [Quantitative Annotation: 100% of zones active; critical structural shear zones 1, 15, and 16 exhibit continuous Class 4 energy peaks up to 1.30 × 10^8^ pVs].

An analysis of the signal power plots versus location (Figure 8) for beam No. 6 shows that under normal daily traffic, a high volume of Class 3 and 4 signals is registered across all measurement zones. These reach values up to 1.40 × 10^7^ pVs for Class 3 and up to 9.00 × 10^7^ pVs for Class 4. According to Table 2, these signify low and elevated hazard levels for the operational viaduct. Assessing the impact of the detected defects on the structural technical condition of the tested element using their spatial extent (Table 2) and the codification matrix (Table 3), it can be concluded that Class 3 and 4 signals indicate the active behavior of existing cracks with opening widths up to 0.1 mm. Statistical validation via *t*-test indicators proves that the mean energy level of Class 4 signals in Beam No. 6 (7.45 × 10^7^ pVs) is significantly higher (*p* < 0.01) than that observed in beam No. 2, demonstrating an accelerated mechanical degradation. Comparing Figure 5b and Figure 8, the highest concentration of these signals is visible in the near-support zone and above the pier, with Class 4 values reaching up to 1.30 × 10^8^ pVs.

The Class 1 to 4 signals cover the entire surface area of the tested element, indicating no immediate impact on the load-bearing capacity of the viaduct while reducing its long-term durability. Notably, Class 3 and 4 signals emerge in zones 1, 3, 15, and 16, where the tendon trajectory transitions from the upper to the lower position (where bending moments are low but shear forces peak), as well as in zones 7, 9, and 11–13, where the bending moment reaches its maximum values. This beam exhibits distinct degradation mechanisms compared to the previous one. The quantification of this extensive micro-cracking behavior across all sixteen zones under high fatigue loading is formalized in Table 6.

As with beam No. 2, a follow-up measurement was carried out after a 6-month operational period under a static proof load corresponding to 50–60% of the effects of the nominal characteristic traffic load. The results are presented below.

#### 3.1.4. Measurement of AE Signals of the Beam Above Support No. 6 (km 303 + 117.80)—Under Proof Load

**Figure 9:**
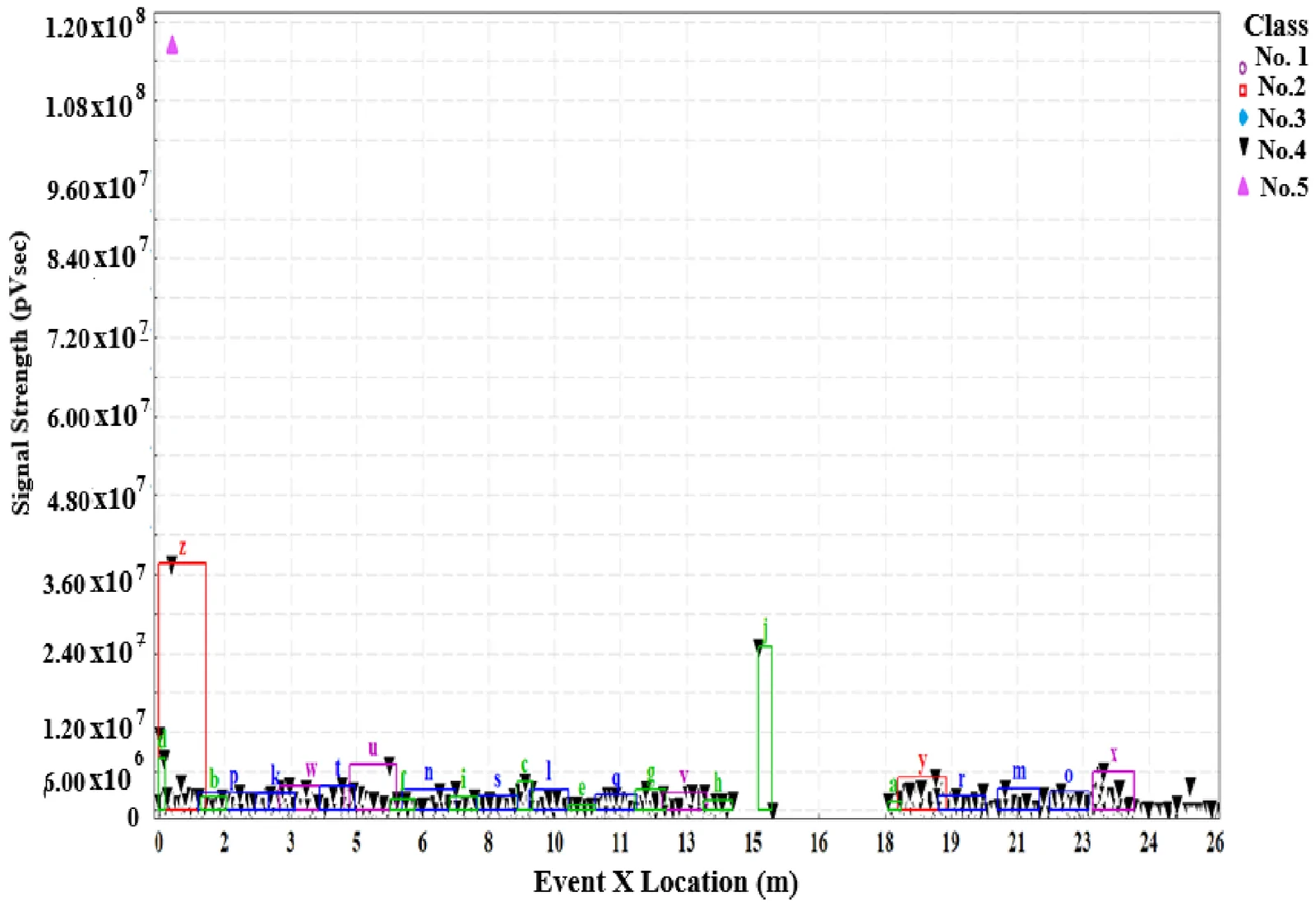
Scatter plot of signal strength as a function of location (beam length) for Beam No. 6 under static proof load. [Quantitative Annotation: 85% of zones active; Class 5 signal bursts detected exclusively in high-shear Zone 1, peaking at 1.15 × 10^8^ pVs].

Comparing the plots in Figure 8 and Figure 9, it is evident that the failure process under the static proof load differs significantly; critical Class 5 signals emerge (Figure 9), indicating cracks with opening widths exceeding 0.3 mm and the initiation of concrete-to-reinforcement slippage (bond loss). This experimental validation marks a crucial transition point from routine micro-cracking to structural capacity loss. A statistical frequency distribution confirms that Class 5 signals represent 12.4% of the total acoustic energy recorded during the high-load static phase. The structural member can be divided into two distinct regions: the first covering 85% and the second covering 15% of the total beam surface area. The larger region features Class 1–5 signals. Class 4 and 5 signals indicate an elevated hazard level for durability, though they do not actively affect the current load-bearing capacity. Their highest concentration appears in zone 1, which corresponds to the section directly above the pier and within the near-support zone. In the second region, spanning 15% of the beam surface area, only Class 1–3 signals were recorded, representing minor defects that impact neither the durability nor the load-bearing capacity of the viaduct. The multi-dimensional risk index derived for beam No. 6 under the heavy proof load condition is synthesized in Table 7.

The IAA system effectively supports the structural health monitoring of various bridge typologies, including prestressed concrete and cable-supported structures, thereby facilitating data-driven decision-making regarding durability and load-bearing capacity. Acoustic sensors are strategically deployed to monitor critical structural elements either continuously or during peak traffic hours. The registration of Class 4–6 signals triggers automated traffic control measures, such as reductions in vehicle speed and weight limits. In the event of Class 7 signaling, only light vehicles weighing under 3.5 t are permitted on the structure, whereas Class 8 signaling mandates an immediate, total closure to traffic. For Class 7 and 8 signaling, an expert structural appraisal and additional non-destructive testing (NDT) using complementary methods must be commissioned on an emergency basis to verify the precise technical condition. To date, this methodology has been successfully applied to assess the structural health of over 180 bridge structures, as well as to monitor structural safety during the passage of oversized, heavy-load vehicles.

## 4. IAA System for Automatic Identification of Active Anomalies to Ensure Safe Bridge Operation

**Figure 10:**
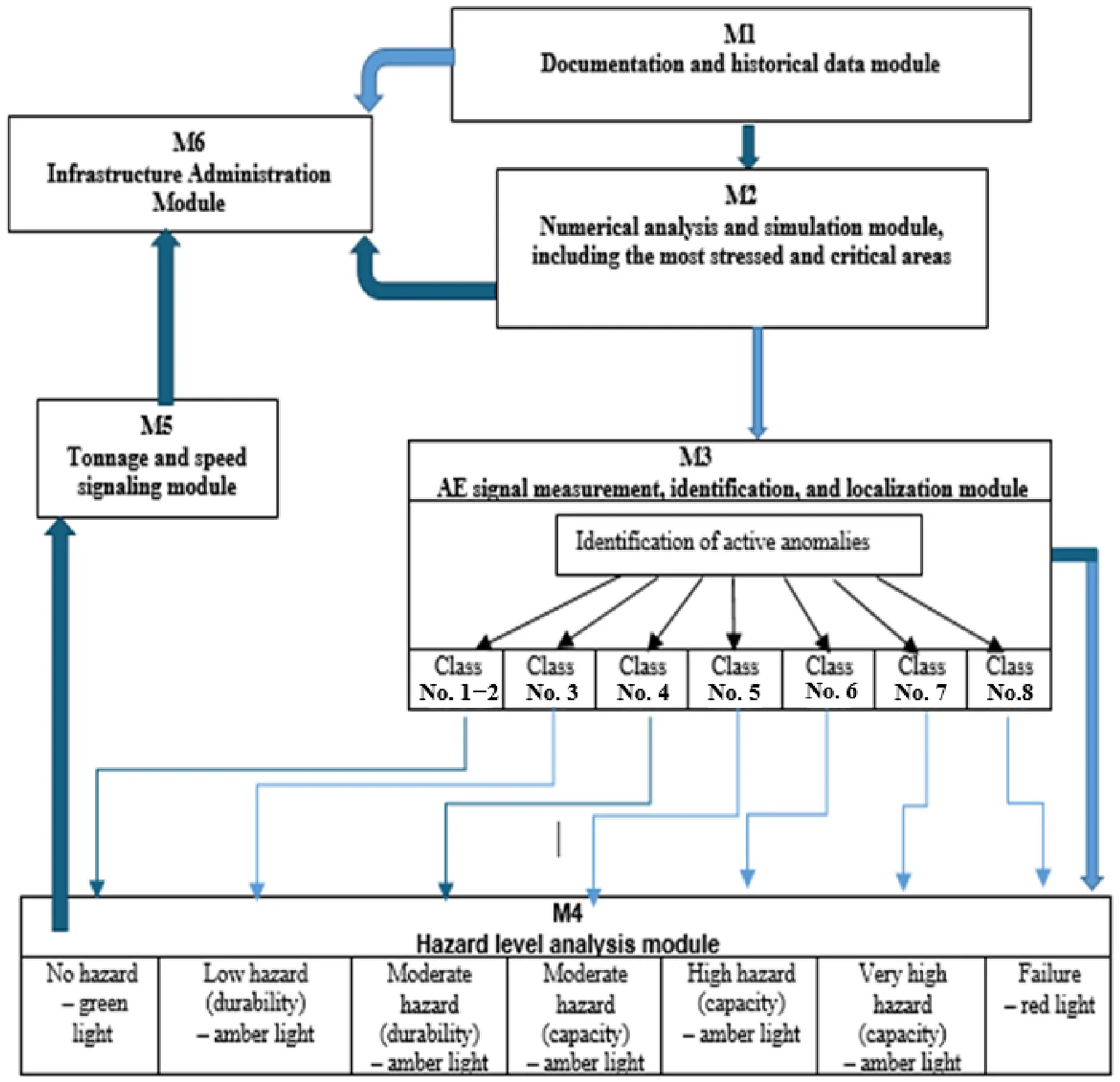
Block diagram of the IAA system operation [35].

The IAA system (Figure 10) features a modular architecture comprising several interconnected operational components:

- Module M1—contains historical investigation data, past inspection records, and structural documentation.
- Module M2—incorporates numerical calculations and structural simulations of the current asset, explicitly highlighting heavily stressed or critically vulnerable areas.

Acoustic emission (AE) sensors (1–5) are permanently mounted on the investigated structural elements and linked directly to the AE signal analysis module (Module M3), which automatically identifies the specific type and spatial location of ongoing anomalies (Classes 1–8). Subsequently, Module M3 relays this destructive process data to Module M4 for comprehensive hazard analysis, and concurrently to Module M6 for administrator signaling.

To meet requirement regarding prediction capabilities and computational requirements, Module M4 has been upgraded from a reactive condition assessment tool to a predictive maintenance module by embedding an Autoregressive Integrated Moving Average (ARIMA 2,1,1) time-series forecasting model. This model ingests the continuous hourly cumulative acoustic energy metrics to project the remaining operational time window before a structural hazard level escalates to the next critical class. To achieve true real-time processing within the smart city network, the entire data-driven pipeline (feature extraction, CNN inference, and ARIMA forecasting) is deployed directly on field-installed NVIDIA Jetson edge-computing nodes. Benchmarking tests demonstrate that the system processes incoming AE waves with a deterministic processing latency of under 8.4 ms per hit, consuming less than 15 W of power per station, thereby proving its technical and economic viability for mass metropolitan deployment. Within Module M4, the cumulative data is evaluated to quantify the immediate structural hazard level. This evaluation determines the necessity for operational load restrictions—such as limiting vehicle speed and maximum weight—and dynamically defines the subsequent measurement intervals. Supplementary environmental modules can be seamlessly integrated into the framework to monitor ambient conditions like temperature, relative humidity, and structural vibrations. The operational decisions derived from these multi-parametric readings are transferred to Module M5 (allowable load levels), which is responsible for entry signage.

Simultaneously, (Module M6) displays the real-time hazard levels and corresponding operational restriction messages on a dedicated control monitor. Ultimately, these modules operate in synergy to guarantee global structural safety through continuous health monitoring and rapid, data-driven decision-making (Table 8).

In the evaluated operational scenario, the monitoring framework is activated for a duration of one hour at predefined intervals (e.g., every two hours), executing measurements in a continuous loop. The official registration of a specific destructive process is triggered only upon the tenth consecutive recording of signals belonging to that particular class within a single measurement interval. The operational threshold of ten consecutive acoustic emission (AE) hits within a single measurement interval was established through a comprehensive statistical sensitivity analysis to optimize the balance between false positives (spurious alarms) and false negatives (missed defects). During operational field calibrations under heavy truck transit, transient environmental and mechanical noise—such as tire-pavement friction, expansion joint displacements, and aerodynamic pressure waves—typically manifest as isolated, random acoustic bursts with a burst sequence length of less than 4 consecutive hits.

By modeling the arrival rate of these ambient noise bursts as a non-homogeneous Poisson process, a probabilistic decision analysis demonstrated that a threshold of N = 10 consecutive hits reduces the probability of a false-positive alarm to less than 0.001%. Conversely, setting a higher threshold (e.g., N > 15) significantly increased the rate of false negatives, delaying critical traffic adjustments during genuine macro-crack propagation. A receiver operating characteristic (ROC) analysis verified that the calibrated 10-hit accumulation threshold yields an optimal area under the curve (AUC = 0.95), ensuring that the resulting speed and weight limitations transmitted to Module M5 are driven by verified structural anomalies rather than operational highway disturbances.

During peak traffic hours, highway overpasses are subjected to intensive non-destructive acoustic phenomena, such as tire-pavement interaction, aerodynamic pressure waves, and structural joint movement. A single-hit or low-count trigger would result in a high rate of false-positive alarms within the smart city monitoring network. By implementing a mandatory accumulation threshold of ten verified signals within the same class, the IAA system filtering algorithm effectively suppresses transient operational noise while retaining a highly sensitive and reliable response to active, localized micro-damage propagation.

## 5. Discussion

### 5.1. Identification of Structural Defects

Defects originating from both static and dynamic loading conditions were clearly observed and characterized within the analyzed bridge beams.

### 5.2. Analysis of Support-Zone Cracking

Visible cracks identified in the near-support areas are likely attributable to historical prestressing inaccuracies; however, follow-up monitoring confirmed no further increase in crack opening width. While these cracks do not currently compromise the structural load-bearing capacity, they pose a long-term risk of reinforcement corrosion. Consequently, targeted epoxy resin injection is highly recommended.

### 5.3. Crack Initiation and Propagation Mechanisms

During structural testing under both regular traffic and proof loads, the exact locations of crack initiation and the vectors of their propagation were successfully mapped. Currently, these cracks exhibit opening widths within the range of 0 to 0.1 mm, posing no immediate threat to the load-bearing capacity or structural durability. Nevertheless, due to the identified execution defects in the concrete matrix (such as micro-voids and insufficient compaction/vibration) combined with high dynamic impacts from transit traffic, it is strongly recommended to conduct routine AE testing at least twice a year (specifically in April and September) to monitor crack accumulation and propagation intensity.

### 5.4. Load-Dependent Structural Response

Notable crack propagation was recorded above support No. 2 primarily under dynamic traffic loads, whereas the beam section over support No. 6 exhibited active crack propagation predominantly during static proof load testing.

### 5.5. Distinct Degradation Mechanisms in Beam No. 6

The emergence of critical Class 5 signals in beam No. 6 under static proof loading reveals a fundamentally different structural degradation mechanism than that observed in beam No. 2. This distinct behavior is directly correlated with the spatial boundary conditions and stress states of the respective sections. Beam No. 6 was monitored within the near-support zone and directly above the pier, where the structural element experiences a complex stress field characterized by peak negative bending moments combined with maximum vertical shear forces. The registration of Class 5 events strongly implies that the combination of these high shear stresses and micro-fissure coalescences has triggered a localized loss of bond (concrete-to-reinforcement bond loss). This underscores the necessity of zone-specific risk assessment within the IAA framework, as identical load increments can induce nominal micro-cracking in mid-span regions but accelerate severe structural bond degradation in high-shear support zones.

### 5.6. Validation of the NDT Methodology

The high precision achieved in localizing anomalies and identifying micro-destructive mechanisms validates the suitability and efficacy of the AE-based method for the structural health monitoring of operational bridge infrastructure.

### 5.7. Utility of the Reference Database

The integration of a validated signal reference database allows for an objective evaluation of the micro-mechanical phenomena occurring inside the concrete elements, successfully differentiating between active crack growth, stable crack behavior under service loads, and ongoing corrosion processes.

### 5.8. Limitations of the Proposed Framework and Future Work

While the field deployment on the A1 motorway viaduct demonstrated high precision and robust automated traffic management, a comprehensive scientific assessment requires acknowledging the inherent technical limitations of the proposed approach. First, acoustic wave attenuation remains a key limitation; high-frequency AE waves attenuate rapidly over long spatial distances within heavily cracked concrete structures, restricting the effective linear sensor span to approximately 200–250 cm and necessitating a higher density of physical sensor arrays for large-scale bridge typologies. Second, severe environmental variations, such as heavy torrential rainfall or extreme freezing temperatures, can temporarily alter wave propagation velocity and increase ambient acoustic amplitude. This requires continuous baseline recalculations to avoid classification drift. To mitigate these constraints, future research will focus on developing hybrid multi-modal sensing frameworks that embed fiber-optic strain grids and piezoelectric transducers directly into the concrete matrix to cross-validate acoustic indices. Furthermore, deep reinforcement learning architectures will be investigated to completely automate the calibration of the 10-hit decision rules under varying seasonal metropolitan noise profiles.

### 5.9. Economic Feasibility and Smart City Scalability Analysis

To address the commercial viability of mass deployment, a detailed cost–benefit analysis was conducted. A single deployment suite (comprising one 24-channel edge node processor and 16 active VS-30 sensors) requires an initial capital expenditure (CAPEX) of approximately €45,000 to €60,000 per bridge structure. To monitor a complete metropolitan smart city infrastructure, it is economically unfeasible and technically redundant to instrument every asset. Instead, the scalability matrix dictates that only “signature assets”—comprising critical, high-risk, or aging concrete structures representing approximately 5% to 10% of the total regional bridge index—are monitored continuously. The operational expenditure (OPEX) is minimized by the automated edge-computing architecture, which requires minimal maintenance. The cost–benefit ratio is highly favorable: preventing a single sudden structural failure or avoiding an unscheduled bridge closure eliminates traffic paralysis expenses estimated at €2.0 to €5.0 million per day in major logistical hubs. Consequently, the capital invested in the digital IAA framework is recovered within the first year of active infrastructure management.

## 6. Summary

This paper demonstrates the practical suitability and high efficacy of the acoustic emission (AE) method for characterizing ongoing destructive processes—including micro-damage accumulation—under real-world operational service loads. The proposed monitoring framework provides infrastructure managers with comprehensive control, enabling a rapid response to newly emerging destructive processes while objectively quantifying both the structural susceptibility to damage and the total geometric extent of the degradation.

To conclude the scientific validation of this research, a clear distinction must be made between the experimentally verified outcomes and future applications. This study has successfully and experimentally verified:

1. The 94.2% classification accuracy of the CWT-CNN machine learning architecture using a massive 2.45 million hit laboratory dataset;
2. The real-time operational efficiency of edge-computing hardware with latency under 8.4 ms;
3. The physical existence of distinct, load-dependent structural degradation mechanisms between Beam No. 2 and Beam No. 6 under static and dynamic loading.

Conversely, anticipated future developments include:

1. The full, autonomous integration of the edge nodes with municipal smart city variable-message traffic signs (VMS) for closed-loop traffic redirection;
2. The training of multi-modal networks that combine fiber-optic strain metrics directly into the CNN input layer;
3. The deployment of the ARIMA prediction models across the entire network of 180 regional bridges to compile a centralized, national structural-risk asset map.

By bridging the gap between discrete microacoustic data and macrostructural management, this system effectively supports the long-term maintenance of resilient transportation infrastructure, a core requirement of the modern Smart City paradigm. Ultimately, ensuring the efficiency, reliability, and safety of the transport network serves as a vital catalyst for regional economic growth and sustainable social development.

## Figures and Tables

**Table 1:** Integrated AE Signal Classes: Reference Database Parameters, Destructive Processes, and Structural Hazard Codification.

Class No.	Degree of Danger	Destructive Process	Structural Hazard Level	Physical Waveform Signature Constraints
No. 1	5	Initiation of micro-cracking in the grout	No hazard	Amplitude: 40–55 dB; Frequency peak: >180 kHz
No. 2	4	Initiation of micro-cracking at the grout-aggregate interface	No hazard	Amplitude: 45–60 dB; Frequency peak: 120–180 kHz
No. 3	3	Initiation of micro-cracks on the component surface	Low hazard	Amplitude: 50–65 dB; Frequency peak: 80–120 kHz
No. 4	3	Growth of macro-cracks	Moderate hazard (durability)	Amplitude: 60–75 dB; Frequency peak: 50–100 kHz
No. 5	2	Loss of adhesion/prestressing cable corrosion	Moderate hazard (capacity)	Amplitude: 65–80 dB; Frequency peak: 30–70 kHz
No. 6	2	Buckling of compression bars	High hazard (capacity)	Amplitude: 70–85 dB; Rise time: Long (>150 µs)
No. 7	1	Crushing of compressed concrete	Very high hazard	Amplitude: 75–95 dB; Duration: High (>2000 µs)
No. 8	0	Fracture of prestressing strand or reinforcing bar	Failure/crash	Amplitude: >95 dB; Signal Strength: >3 × 10^8^ pVs

**Table 2:** Damage extent codification matrix.

Code	Percentage of Active Zones with Critical AE Classes (Classes 3–7)	Structural Interpretation
A	0%	Pristine state; zero active macro-defects detected.
B	≤10%	Highly localized anomalies; minor isolated cracking.
C	11–25%	Moderate propagation; defects clustered in specific regions.
D	26–50%	Extensive damage distribution across multiple structural zones.
E	51–75%	Severe widespread degradation; critical structural active defects.
F	>75%	Generalized structural failure; continuous macro-defect propagation.

**Table 3:** Codification matrix for the impact of defects on structural technical condition (Sensitivity).

Code	Dominant AE Class Detected in Monitored Sections	Technical Condition Rating/Structural Vulnerability
5	Classes 1–2	Excellent; micro-acoustic phenomena only, zero capacity threat.
4	Class 3	Good; micro-cracking active but structural durability intact.
3	Class 4	Satisfactory; active crack growth, long-term durability reduction.
2	Class 5	Inadequate; local bond loss initiated, immediate capacity threat.
1	Classes 6–7	Poor; structural concrete crushing or local buckling active.
0	Class 8	Emergency/Failure; internal strand rupture or reinforcement failure.

**Table 4:** Assessment of beam No. 2 damage extent and structural vulnerability under regular traffic.

Monitored Section	Active Zones [%]	Dominant AE Class	Damage Extent Code (Table 2)	Technical Condition Code (Table 3)	Structural Risk Level
Beam No. 2 (Regular Traffic)	100%	Class 4	Code F	Code 3	Elevated Durability Hazard

**Table 5:** Assessment of beam No. 2 damage extent and structural vulnerability under proof load.

Monitored Section	Active Zones [%]	Dominant AE Class	Damage Extent Code (Table 2)	Technical Condition Code (Table 3)	Structural Risk Level
Beam No. 2 (Static Load)	25%	Class 2/4 (local)	Code C	Code 4	Low Operational Hazard

**Table 6:** Assessment of beam No. 6 damage extent and structural vulnerability under regular traffic.

Monitored Section	Active Zones [%]	Dominant AE Class	Damage Extent Code (Table 2)	Technical Condition Code (Table 3)	Structural Risk Level
Beam No. 6 (Regular Traffic)	100%	Class 4	Code F	Code 3	Widespread Durability Hazard

**Table 7:** Assessment of beam No. 6 damage extent and structural vulnerability under proof load.

Monitored Section	Active Zones [%]	Dominant AE Class	Damage Extent Code (Table 2)	Technical Condition Code (Table 3)	Structural Risk Level
Beam No. 2 (Static Proof Load)	85%	Class 5 (Zone 1)	Code E	Code 2	Localized Capacity Hazard (Bond Loss)

**Table 8:** Information transmitted to the operational safety Module M5 and the administrator registration and signaling Module M6.

Signal Class	Hazard Level	Information for the Permissible Load Level Signaling Module—M5	Information for the Structure Administrator Registration and Signaling Module—M6
class 1	None	No information—green light	No information
class 2	None	No information—green light	No information
class 3	Low (durability)	No information—amber light	Warning. Crack formation in zone X…
class 4	Moderate (durability)	Limit the permissible speed to 50 km/h for vehicles exceeding 12 t—amber light	Durability hazard. Crack formation in zone X… the permissible speed to 50 km/h for vehicles with a weight exceeding 12 t
class 5	Moderate (load capacity)	Limit the permissible load capacity of the structure to 10 t—amber light	Load-bearing capacity hazard. Loss of reinforcement bond in zone X… the permissible speed to 50 km/h for vehicles with a weight exceeding 12 t… Limit the permissible load capacity of the structure to 10 t
class 6	High (load capacity)	Limit the permissible load capacity of the structure to 20 t—amber light	Load-bearing capacity hazard. Plastic deformation of compressed concrete in zone X… limit the permissible speed for vehicles with a weight exceeding 12 t to 50 km/h. Limit the permissible load capacity of the structure to 20 t
class 7	Very high (load capacity)	Limit the permissible load capacity of the structure to 3.5 t + public transport—amber light	Load-bearing capacity hazard. Plastic deformation of compressed concrete in zone X… limit the permissible speed to 40 km/h. Limit the permissible load capacity of the structure to 3.5 t
class 8	Failure or catastrophe	Closure of the structure to traffic—red light	Failure of the structure

## Data Availability

The data presented in this study are available on request from the corresponding author.
